# The relationship between cadmium exposure and preeclampsia: a systematic review and meta-analysis

**DOI:** 10.3389/fmed.2023.1259680

**Published:** 2023-12-01

**Authors:** Chu Li, Jiamin Luo, Yunping Yang, Qianqian Wang, Yanmei Zheng, Zixing Zhong

**Affiliations:** ^1^Department of Obstetrics, Center for Reproductive Medicine, Zhejiang Provincial People’s Hospital (Affiliated People’s Hospital, Hangzhou Medical College), Zhejiang, China; ^2^The Second Clinical College of Zhejiang Chinese Medical University, Hangzhou, China; ^3^Department of Ultrasound Medicine, Center for Reproductive Medicine, Zhejiang Provincial People’s Hospital (Affiliated People’s Hospital, Hangzhou Medical College), Zhejiang, China

**Keywords:** Cadmium, heavy metal, preeclampsia, blood pressure, systematic review, meta-analysis

## Abstract

**Background:**

Cadmium (Cd) is a heavy metal associated with several human disorders. Preeclampsia is a major cause of maternal mortality worldwide. The association between maternal Cd exposure and preeclampsia remains elusive.

**Methods:**

To better understand this relationship, we conducted a systematic review and meta-analysis of eligible studies from five databases (PubMed, Embase, Web of Science, Scopus, and CNKI) from their inception to September 10, 2022. The quality of these studies was evaluated using the Newcastle-Ottawa quality assessment scale (NOS). We use random-effects models to calculate overall standardized mean differences (SMDs) and 95% confidence intervals (CIs). Sensitivity analyses were performed to assess the robustness of our results. We also evaluated publication bias using Egger’s and Begg’s tests. Additionally, we conducted meta-regression and sub-group analyses to identify potential sources of heterogeneity between studies.

**Results:**

Our analysis included a total of 17 studies with 10,373 participants. We found a significant association between maternal cadmium exposure and the risk of preeclampsia (SMD 0.27, 95% CI 0.09–0.44, *p* < 0.01). No significant publication bias was detected in Begg’s or Egger’s tests. Meta-regression suggested that geographical location, year of publication, cadmium samples, sample size, and measurement methods did not contribute to heterogeneity between studies.

**Conclusion:**

Our findings suggest that maternal blood cadmium levels are associated with an increased risk of preeclampsia. In contrast, the pregnant women’s urine or placental levels of cadmium may not suggest preeclamptic risk during pregnancy. Further high-quality clinical studies and animal experiments are needed to understand this association better.

**Systematic review registration:**

PROSPERO, https://www.crd.york.ac.uk/prospero/display_record.php?RecordID=361291, identifier: CRD42022361291.

## 1 Introduction

Cadmium (Cd) is a natural element usually exists in soil, rocks, coal, and mineral fertilizer (1). As a heavy metal, it can co-exist with zinc, copper, and lead minerals. Cadmium is widely used in the mining and smelting of non-ferrous metals, the manufacture of fertilizers, and the burning of fossil fuels and wastes. Human exposure to Cd is primarily via taking contaminated food, especially leafy green vegetables. Spinach (0.124 mg/kg) and lettuce (0.051 mg/kg) contain the highest levels of Cd. Regular consumption of shellfish and animal organs (e.g., liver and kidney) can increase the risk of cadmium exposure (2). According to the World Health Organization and the International Agency for Research on Cancer, Cd is the group I human carcinogen (3). Epidemiological studies have shown that cadmium exposure via respiration or drinking contaminated water could lead to cancer of the lung (3, 4), prostate (3), kidney (3), and bladder (5).

Preeclampsia (PE) is a major feto-maternal threat that affects around 5% of pregnancies worldwide (6–9). It is defined as either a systolic blood pressure (BP) of 140 mmHg or more or a diastolic blood pressure of 90 mmHg or more, or both, on two occasions at least 4 h apart after 20 weeks of gestation in a previously normotensive woman (10). Apart from hypertension, preeclampsia also involves multiple systemic presentations, including reduced maternal platelets, headache, and fetal growth restriction, etc. This has caused global concerns, imposing a heavy burden on public health. Despite multiple genetic, angiogenic, and immune predispositions identified in recent decades, its etiology remains unclear (11).

The relationship between cadmium and preeclampsia has not been fully explored before, but we may extrapolate it from existing studies focusing on the association between Cd exposure and varieties in BP. Gallagher et al. were the first to investigate the association between cadmium exposure and hypertension systematically (12). They identified a positive correlation between blood Cd and BP and a negative correlation between urine Cd and BP. These were more prominent among females. More recent studies have explored the intrinsic role of Cd in the development of vasculopathy, eventually leading to hypertension (12–15). Possible mechanisms of cadmium-induced hypertension can be renal failure, calcium signaling disruption, oxidative stress disorder, obstruction of the renin-angiotensin system, and vascular endothelial disorder (16, 17).

Reproductive age women have notably higher blood levels of Cd than men (median 0.41 vs. 0.17 ug/L, *p* < 0.01) (18, 19). Whether increased levels of Cd predispose them to developing preeclampsia has not been investigated. We only know that the placenta is an effective barrier against Cd, with only 0.01% of maternal Cd passed on to fetuses (20). Cd is hence partially concentrated in the placenta, causing potential placental and feto-maternal damage, such as fetal growth restriction (FGR), and maternal gestational diabetes mellitus (GDM) (21).

To our knowledge, we are the first to conduct an updated systematic review to focus specifically on the relationship between Cd exposure and PE.

## 2 Methods

The study protocol was registered with PROSPERO (No. CRD42022361291). The article was performed following the Preferred Reporting Items for Systematic Reviews and Meta-Analyses (PRISMA) guidelines (22).^1^

### 2.1 Search strategy

Two investigators (ZX.Z. and C.L.) independently searched four international electronic databases (PubMed, Scopus, Embase and Web of Science) as well as the China National Knowledge Infrastructure (CNKI) from database inception to September 10, 2022. The search syntax applied in the databases was (Cadmium OR “heavy metal” OR “trace element”) AND (preeclampsia OR eclampsia OR pre-eclampsia OR gestosis OR “pregnancy hypertension” OR “pregnancy-induced hypertension” OR “pregnancy-associated hypertension” OR “hypertensive disorders of pregnancy” OR “pregnancy toxemia” OR “gestational hypertension” OR “HELLP syndrome*”). The detailed search strategies can be accessed in Supplementary Search Strategy. Two independent reviewers (C.L. and ZX.Z.) initially screened all articles to assess for eligibility by the title and abstract. In addition, we searched for a list of the references and related review articles for additional articles. These include studies written in English or Chinese.

### 2.2 Eligibility criteria and study selection

The criteria for inclusion and exclusion of studies were established prior to the literature search. Articles were included if the following criteria were met: (1) observational studies involving maternal levels of cadmium and preeclampsia; (2) the exposure of interest should include cadmium while the outcome of interest should include preeclampsia; (3) the control should be pregnant women instead of non-pregnant women; (4) investigated the relationship between exposure to cadmium and any results mentioned in the search statement. These studies were included regardless of the age range, gravidity, parity and singleton/ multiple pregnancies of participants.

Articles were excluded if: (1) Studies not based on Cd level in maternal serum or placenta or urine, for instance, Cd exposure level in soil, or Cd level in maternal hair. (2) Duplicates, irrelevant studies, letters, reviews, commentaries, and conference abstracts were excluded.

Selected articles were retrieved thoroughly and further assessed for eligibility. ZX.Z. and C.L. screened the studies, and a third reviewer, JM.L. was to resolve any discrepancies between the two.

### 2.3 Data extraction and quality assessment

The following information was extracted by two researchers (ZX.Z. and C.L.) from the studies included: the first author and year of publication, location of the study, study types, number of participants, types of specimen, sample size, Cd level, average maternal age with standard deviation, sample collect time, methods of measurement and diagnostic criteria.

The quality of case–control and cohort studies was evaluated by the Newcastle-Ottawa Scale (NOS). A nine-star rating system evaluated by three dimensions, such as selection, comparability, outcome ascertainment. A score between 7 and 9 indicates good quality, while 4 to 6 was considered moderate quality. Poor quality was defined if the score was ≤3. Cross-sectional studies were assessed by a modified form of NOS, scoring zero to ten. Seven or more suggests a good quality, four to six indicates fair quality, while poor quality is considered for studies being score three or less (23).

### 2.4 Sub-group analysis and meta-regression analysis

Sub-group analysis and meta-regression were performed to assess whether sample types (blood, urine, placenta), geographic locations, year of publication, or type of measurement or sample size influenced the relationship. We divided all studies into five groups based on the original location of the study population: Asian studies were all from China. Studies from the USA were allocated to the American group. African studies consisted of reports from the Democratic Republic of Congo and South Africa. Middle-East studies covered reports from Iran and Turkey. European studies contained reports from France and the Republic of Serbia.

### 2.5 Statistical analysis

We calculated the results and performed data analysis via Review Manager 5.1 (The Nordic Cochrane Center, Copenhagen, Denmark) and Stata version 16.0 (StataCorp, College Station, TX, United States). The standardized mean differences (SMDs) with 95% confidence intervals (CIs) were used to summarize maternal cadmium exposure levels. Heterogeneity was tested by *I*^2^ (*I*^2^ ≥ 50% indicates high heterogeneity). The forest plot was used to visualize the overall results, with the random-effect model (REM) being adopted for calculation as the heterogeneity was considered significant. A sensitivity analysis was performed, removing each study once to assess whether any study influenced the overall results. Publication bias was visualized via funnel plot and verified by Begg’s and Egger’s tests.

## 3 Results

### 3.1 Study selection

**Figure 1:**
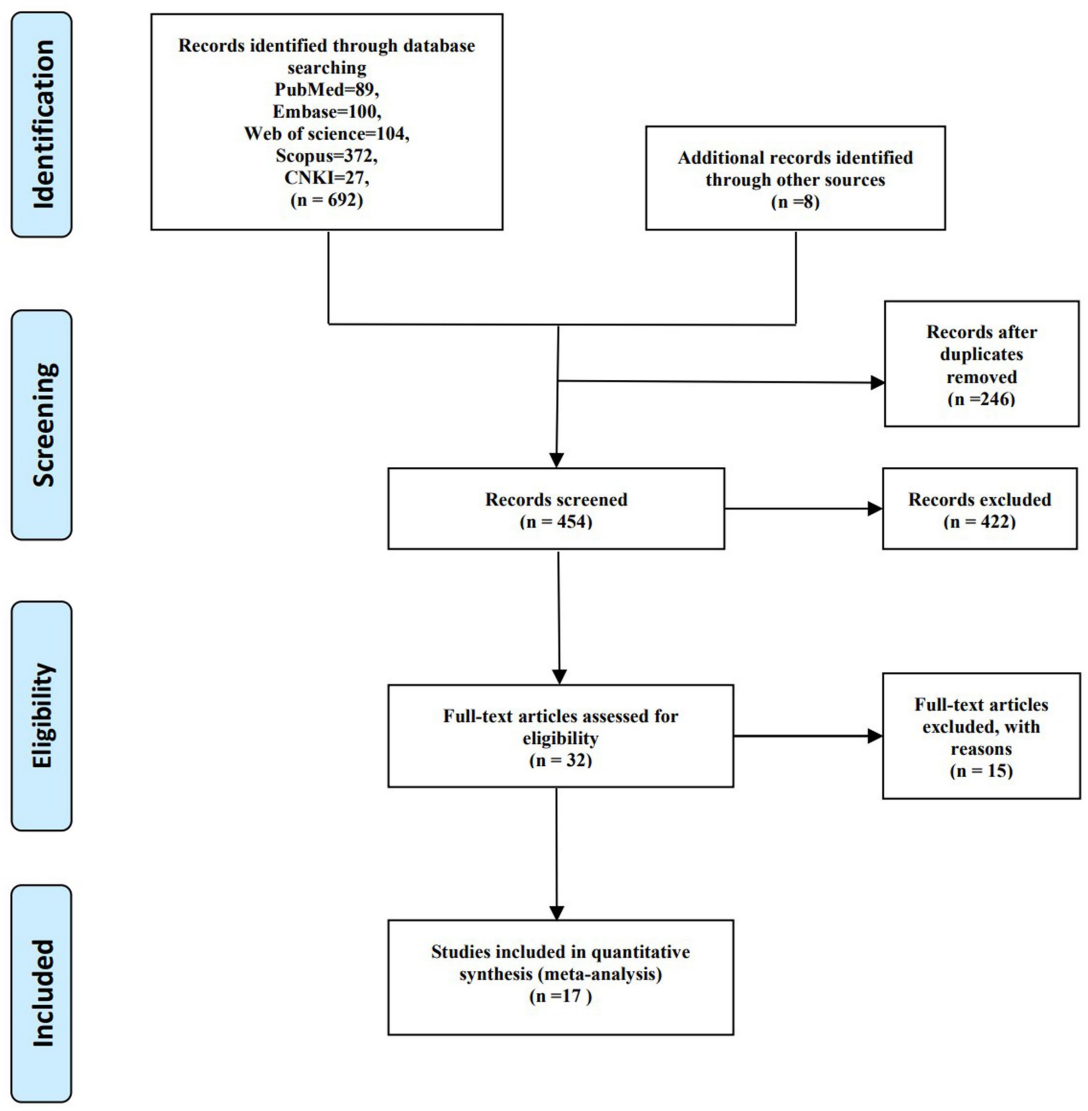
PRISMA flow diagram for study selection process on correlation between maternal cadmium exposure and preeclampsia.

Six hundred and ninety-two articles were identified via searching five databases (PubMed, Scopus, Embase, Web of Science, and CNKI). After examining all the references from full-text articles, eight additional studies were identified. Of the total 700 studies, 246 were deleted due to duplication. Four hundred and fifty-four literatures were further screened, and 422 were excluded after preliminarily browsing the title and abstract. Thirty-two records were being evaluated in the full-text assessment. Fifteen of them were removed for reasons: Six studies were excluded for not reporting cadmium samples (24–29). Four that reported amniotic fluid or nail samples were not included in our study (30–33). One article was excluded for overlapping the study population with another article, which was included (34). One was a conference paper (35). Two studies reported incomplete or absent data, such as no integral data for normal pregnancy and PE (36, 37). One article failed to make it into the finalists as it mainly focused on the relationship between Cd exposure and obstetric outcomes during pregnancy without preeclampsia (38). The remaining 17 reports were included in a systematic review and meta-analysis (39–55). Details of the study selection process can be illustrated in Figure 1.

### 3.2 Characteristics of included studies

The 17 articles from 9 countries spanning over two decades. The size of the population between different studies was contrastingly different, ranging from 46 (minimum) to 5,429 (maximum). Differences were also observed in varied types of samples (blood, urine, placenta) and methods of measurement, such as inductively coupled plasma optical emission spectrometry (ICP-OES), inductively coupled plasma mass spectrometry (ICP-MS), and atomic absorption spectrometry (AAS). Despite these differences, most studies adopted ACOG’s diagnostic criteria for preeclampsia. More detailed information can be seen in Table 1.

**Table 1:** Characteristics of included studies.

Study	Nation	Design	Age of PE		Age of control		Cd of PE	Cd of control	Sample type (unit)	Methods of measurement	diagnostic criteria of PE
			Mean ± SD	*N*	Mean ± SD	*N*	Mean ± SD	Mean ± SD			
Laine et al. (44)	US	NCC	24.0 ± 6.0	86	25.0 ± 6.3	86	3.7 ± 3.4	3.5 ± 2.1	Placental (ng/g)	ICP-MS	ACOG
Wang et al. (49)	China	CC	26.3 ± 4.0	51	27.0 ± 2.9	51	1.2 ± 0.8 4.3 ± 2.0 0.3 ± 0.2	1.1 ± 0.4 3.6 ± 3.6 0.4 ± 0.2	Blood (μg/L) Placental (μg/kg) UCB (μg/L)	ICP-MS	ACOG
Bommarito et al. (51)	US	CC	33.0 ± 4.5	28	32.7 ± 4.9	355	0.1 ± 0.1	0.1 ± 0.1	Urine (μg/L)	ICP-MS	ACOG
Li et al. (55)	China	CC	31.2 ± 8.1	23	32.4 ± 3.3	23	0.6 ± 0.6 15.1 ± 1.7	0.4 ± 0.4 11.2 ± 1.1	Blood (μg/dL) Placental (ng/g)	ICP-MS	ACOG
Ovayolu et al. (54)	Turkey	CC	30.6 ± 7.7	46	28.0 ± 6.6	46	0.6 ± 0.8	0.5 ± 0.6	Blood (μg/L)	ICP-MS	ACOG
Wang et al. (53)	China	CC	NS	427	NS	427	1.1 ± 2.4	1.2 ± 5.3	Blood (μg/L)	ICP-MS	(56)
Liu et al. (52)	US	CS	29.1 ± 6.2	115	28.0 ± 6.3	1,159	0.8 ± 0.4	0.7 ± 0.4	Blood (μg/L)	ICP-MS	ACOG
Liu et al. (47)	China	CS	29.4 ± 4.1	199	28.5 ± 3.7	5,230	0.6 ± 2.7	0.4 ± 2.0	Urine (μg/L)	ICP-MS	(57)
Maduray et al. (46)	South Africa	CC	25.0 ± 5.0	43	24.0 ± 5.0	23	0.1 ± 0.0 4.0 ± 0.9	0.1 ± 0.3 3.8 ± 0.6	Blood (μg/L) Hair (ng/g)	ICP-OES	(58)
Elongi Moyene et al. (45)	DR Congo	CC	27.1 ± 6.1	88	26.7 ± 5.9	88	2.1 ± 2.4	0.5 ± 0.3	Urine (μg/L)	ICP-MS	(59)
Wang et al. (42)	China	CC	NS	10	NS	88	5.7 ± 4.2 1.0 ± 6.1	4.8 ± 4.2 1.0 ± 6.4	Blood Urine (μg/L)	AAS	NS
Kolusari et al. (41)	Turkey	CC	27.9 ± 5.2	47	27.9 ± 4.3	48	0.0 ± 0.0	0.0 ± 0.0	Blood (μg/dL)	ICP-OES	ACOG
Vigeh et al. (40)	Iran	CC	26.0 ± 4.0	31	26.9 ± 5.7	365	0.5 ± 0.3 0.3 ± 0.4	0.5 ± 0.3 0.4 ± 0.4	Blood UCB (μg/L)	ICP-MS	ACOG
Zhang et al. (50)	China	CC	28.6 ± 2.0	40	27.7 ± 2.2	40	38.3 ± 11.4	18.5 ± 6.2	Blood (μg/L)	ICP-MS	ACOG
Yazbeck et al. (43)	French	Cohort	NS	106	NS	865	0.9 ± 0.5	0.9 ± 0.6	Blood (μg/L)	AAS	(60)
Kosanovic et al. (39)	Serbia	CC	NS	23	NS	37	1.5 ± 0.5 0.4 ± 0.1	1.3 ± 0.9 0.3 ± 0.1	Blood UCB (μg/L)	AAS	NS
Musa Obadia et al. (48)	DR Congo	CC	30.6 ± 6.4	40	31.4 ± 4.7	40	0.7 ± 0.4 3.5 ± 5.1	0.7 ± 0.2 0.7 ± 0.5	Blood Urine (μg/L)	ICP-MS	NS

NS, not stated; NCC, Nested case-control; CC, Case-control study; CS, Cross sectional; PC, Prospective cohort.

ICP-MS, Inductively coupled plasma mass spectrometry; ICP-OES, Inductively coupled plasma optical emission spectrometer; AAS, Atomic absorption spectrophotometry; UCB, Umbilical cord blood.

### 3.3 Results of the systematic review

The 17 studies were further divided into 14 case–control or nested case–control, two cross-sectional and 1 cohort studies, and assessed by NOS. Since their inclusion and exclusion criteria are similar, we combine them to increase the sample size. One article was assessed as moderate quality and none of the studies was evaluated as poor quality. The specific score is accessible in Supplementary Tables S1–S3.

### 3.4 Results of meta-analysis

**Figure 2:**
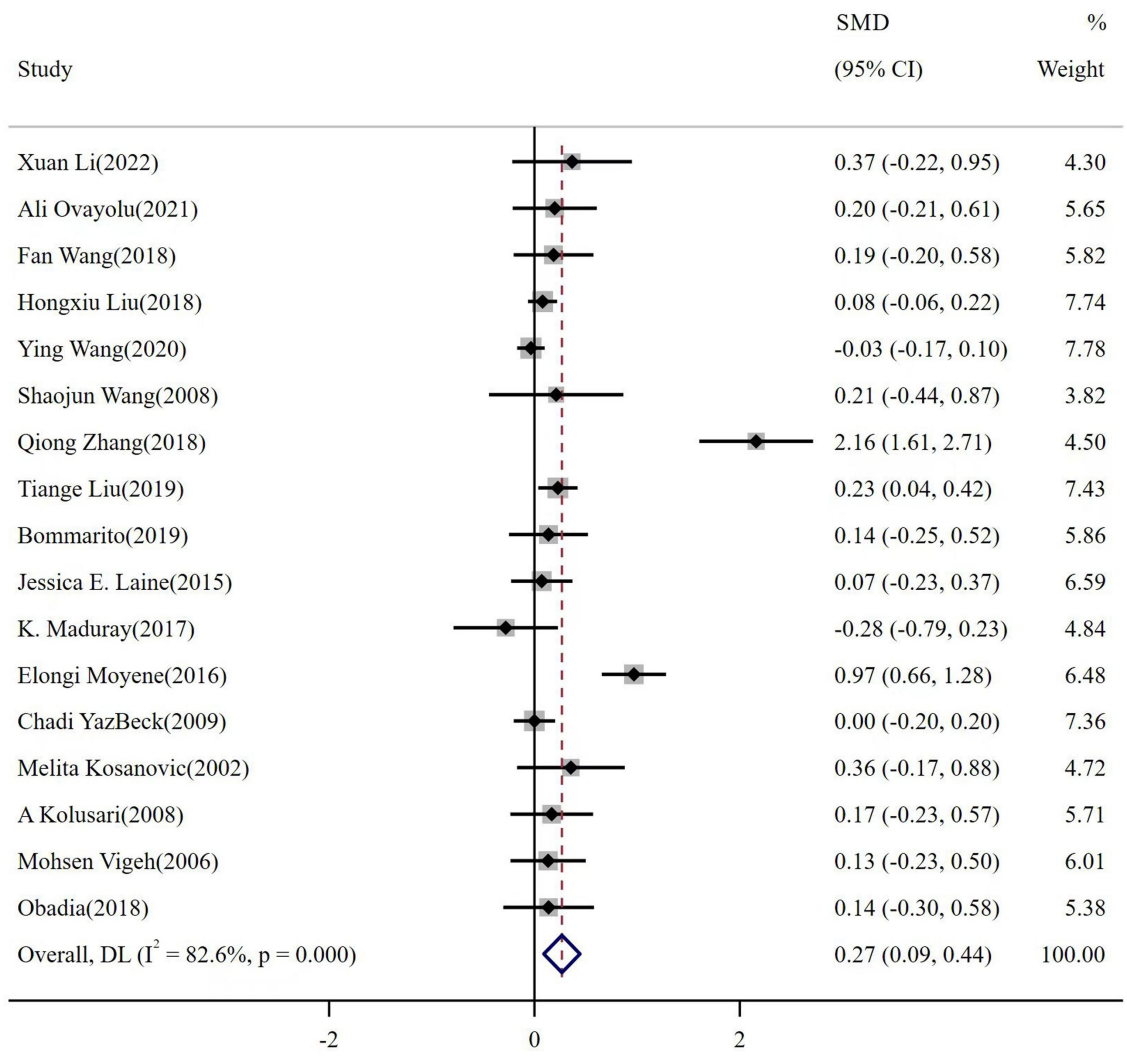
The forest plot of correlation between maternal cadmium exposure levels in preeclamptic and healthy pregnant women.

**Figure 3:**
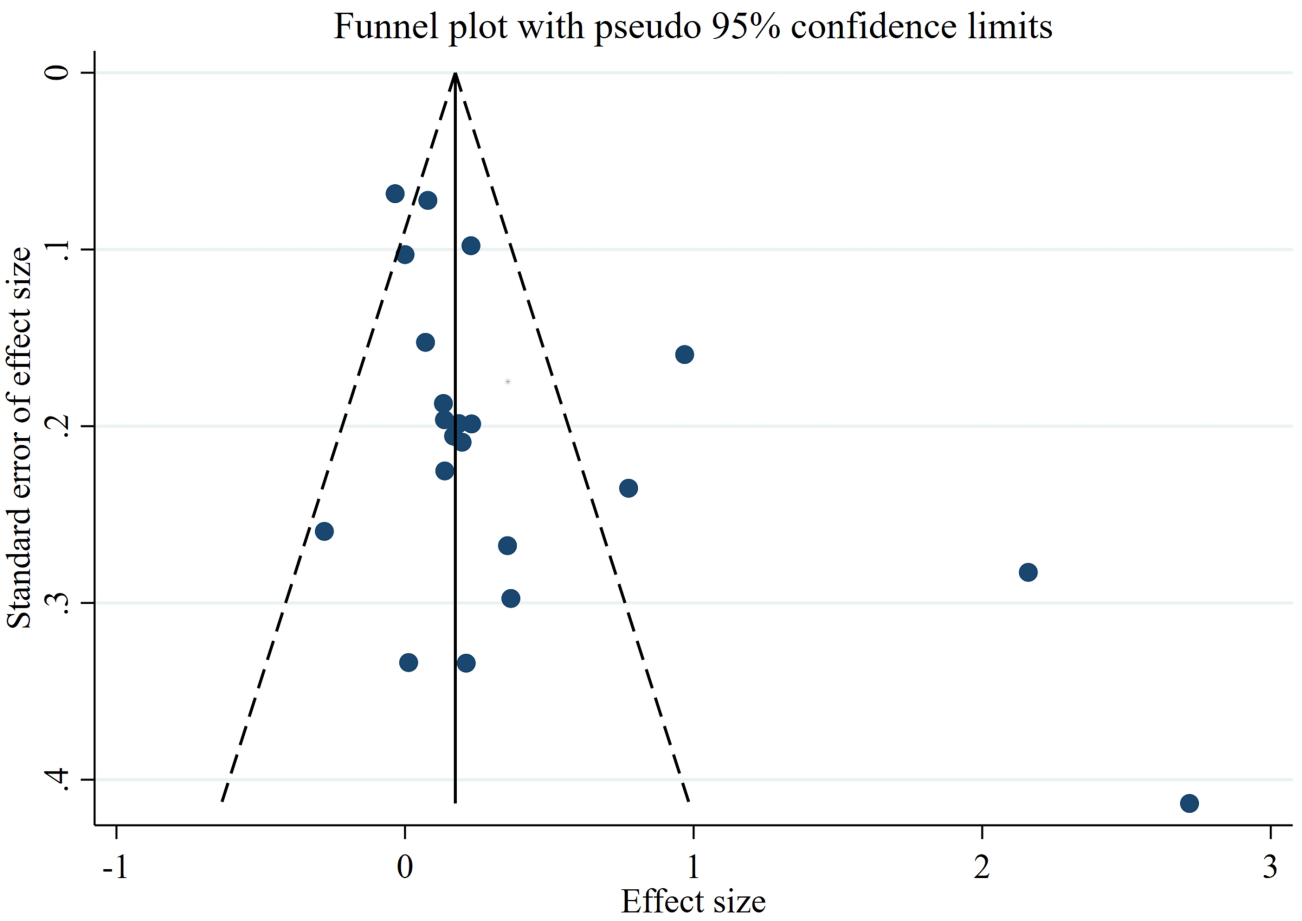
The funnel plot to assess publication bias.

The meta-analysis of the 17 reports included a total of 10,373 participants. The number of healthy pregnant controls was much higher than the case group. There were 1,403 preeclamptic women, and a six-fold group of non-preeclamptic pregnant women (1,403 vs. 8,970). The overall results showed that maternal cadmium exposure in preeclamptic women was significantly higher than that of healthy pregnant control (SMD 0.27, 95% CI 0.09–0.44); (*I*^2^ = 82.6%; *p* < 0.01), see Figure 2. After systematic assessment, we extracted the variables associated with maternal Cd exposure to pool the overall results. The funnel plot showed insignificant publication bias, as shown in Figure 3. Publication bias was evaluated quantitatively by Begg’s test and Egger’s test (*z* = 2.06, *p* = 0.039; *t* = 1.86, *p* = 0.082; see Supplementary Figure S1). The leave-one-out sensitivity analysis showed that Moyene et al. and Zhang et al. reported inverse contributions to the combined results (Supplementary Figure S2) (45, 50).

### 3.5 Results of sub-group meta-analysis

**Figure 4:**
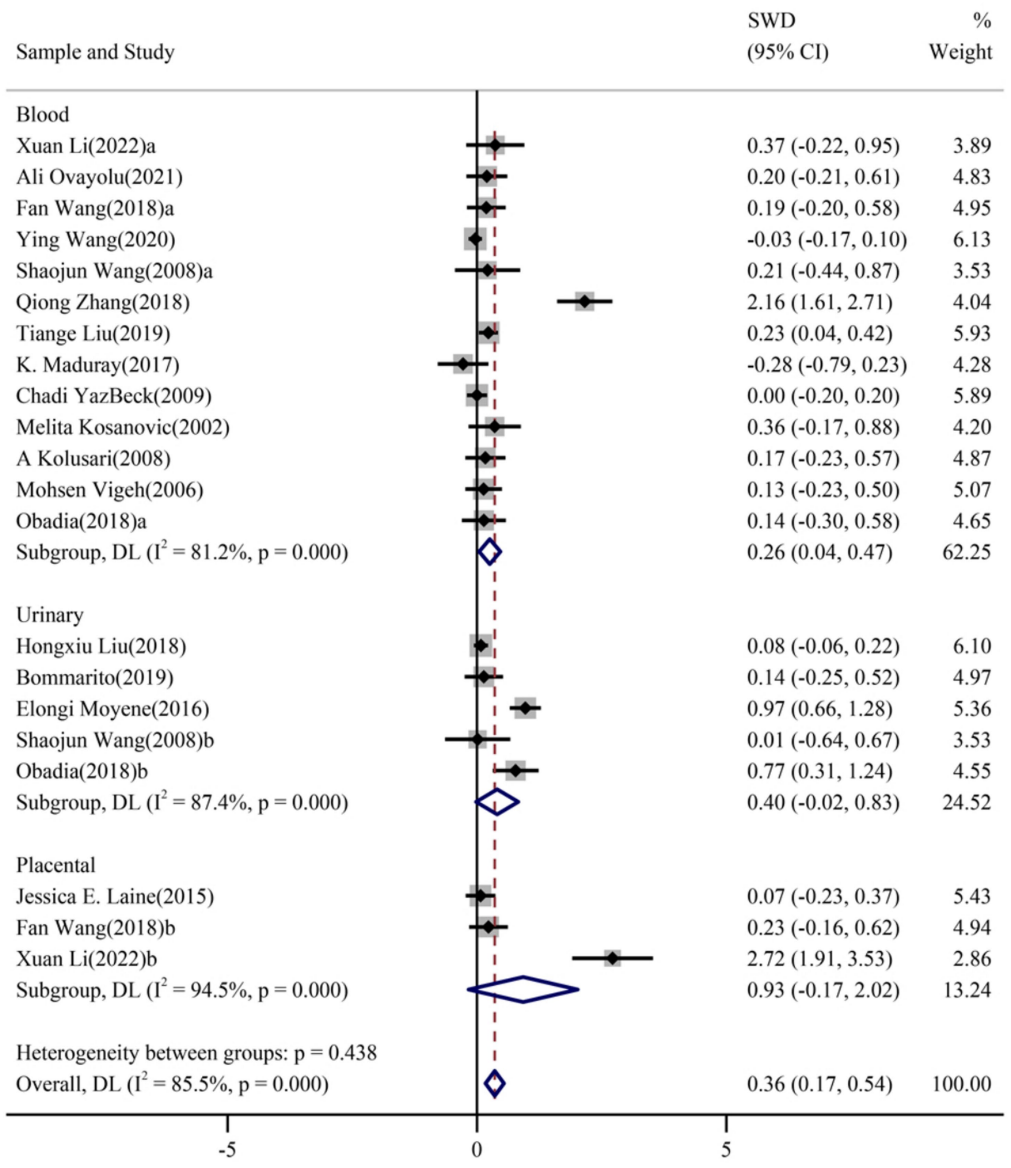
Forest plots of the combined associations of cadmium with PE stratified by types of samples of cadmium.

We performed subgroup analysis to further identify any contributing factors that lead to the heterogeneity between studies. Thirteen studies involved maternal blood Cd levels between preeclamptic and normotensive were allocated in the same sub-group. Five records provided maternal urine cadmium levels, while three showed the placental levels of Cd in the two populations. The pooled results demonstrated that maternal blood cadmium levels were associated with an increased risk of preeclampsia (SWD_BCd_ = 0.26; 95% CI: 0.04–0.47, Figure 4). By contrast, no significant association was found in maternal urine or placental Cd levels (SWD_UCd_ = 0.40; 95% CI: −0.02 to 0.83; SWD_PCd_ = 0.93; 95% CI: −0.17 to 2.02; respectively, Figure 4).

### 3.6 Results of meta-regression

**Figure 5:**
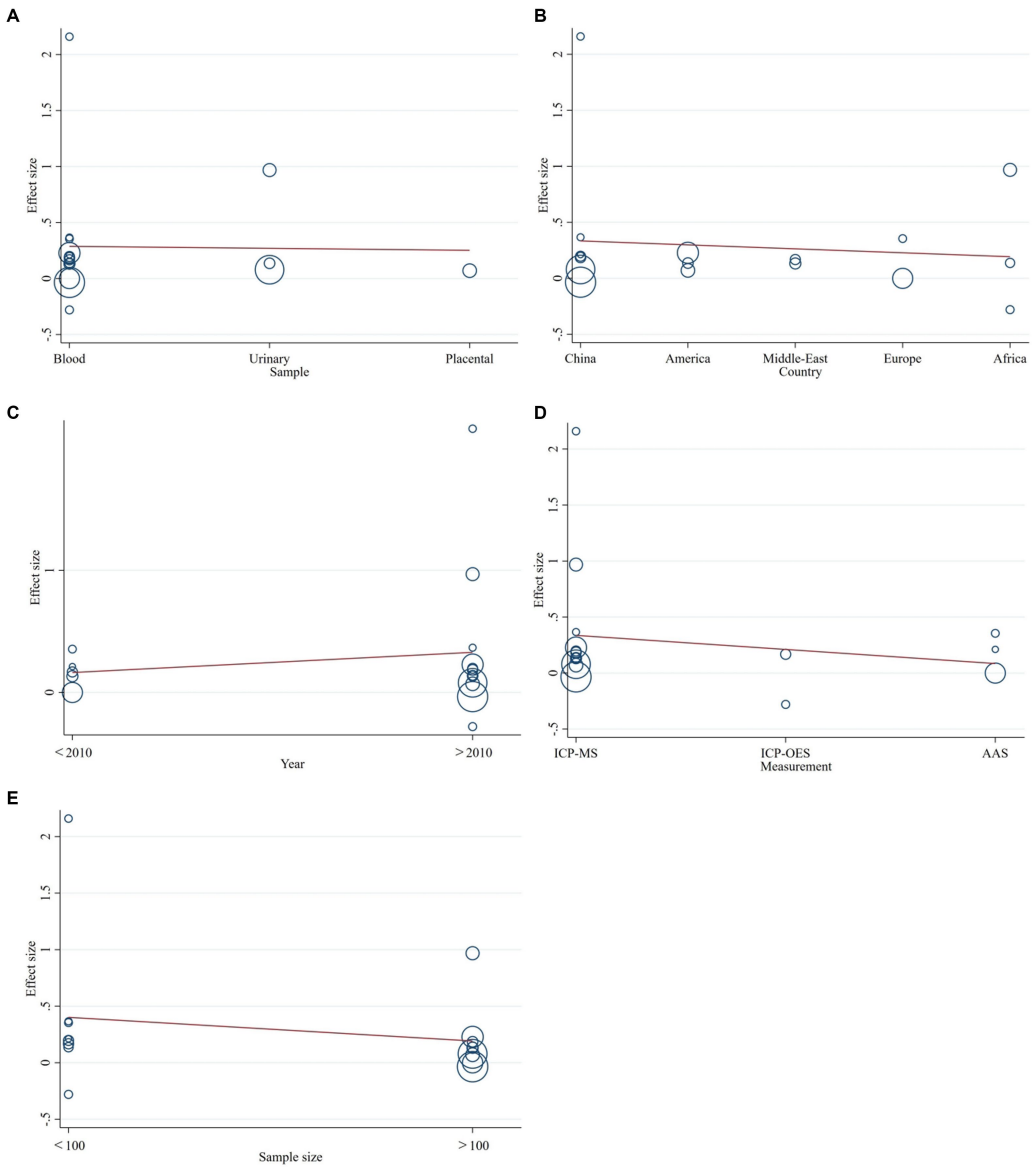
The meta-regression to assess publication bias. **(A)** types of samples of cadmium, **(B)** country of the study, **(C)** year of publication, **(D)** measurement methods, or **(E)** sample size.

Meta-regression was performed because of significant heterogeneity between studies. Different year of publication, geographical locations, different types of samples of cadmium, sample size, and measurement methods were further tested to look for potential causes of heterogeneity. However, it turned out that none of them was the major contributor (*p*-value of sample type: 0.139; *p*-value of location: 0.465; *p*-value of measurements: 0.685; *p*-value of year: 0.609; *p*-value of sample size: 0.145). Detailed information can be seen in Figure 5.

## 4 Discussion

In the systematic review and meta-analysis, we recruited 17 studies involving 10,373 pregnant women to confirm the positive association between Cd exposure and preeclampsia. We also observed that sample selection bias, measurements, study designs may not contribute to the heterogeneity between studies. The meta-analysis found that maternal cadmium exposure in preeclamptic women was significantly higher than that of healthy pregnant controls. Generally, blood Cd usually reflects recent Cd exposure, while urine Cd levels generally reflect long-term Cd exposure (2, 61). The evidence linking maternal Cd exposure with preeclampsia implies that reproductive-age women should stop smoking and choose their diet carefully, avoiding potentially contaminated foods, particularly leafy vegetables, potatoes and grains, peanuts, and soybeans. Most of which are popular food choices in daily life. Due to the long process of Cd metabolism, the preparation should be as early as a couple’s family planning (62, 63).

The correlation between Cd and hypertensive disorders was first proposed by Henry. In his animal study in 1965, hypertensive animal models were successfully replicated through injection into pregnant rats with a water solution of Cd (64). The pregnant rats developed some forms of typical clinical manifestations, such as hypertension, albuminuria and FGR during pregnancy. Autopsy histopathological report revealed endothelial cells swelling, thickening of the media of the renal vessel walls, and protein tube formation in the renal tubules. The impaired placental angiogenesis was the typical change in human with PE (34, 65). Cd can change the placental structure, thereby affecting the function of placental transfer, predisposing pregnant women to PE (66).

Cadmium can also express a potent estrogen-like activity *in vivo*, stimulating the growth of myometrium (67, 68). In animal models, exposure to Cd can increase the weight of uterus and induce hormone-regulating gene expression even after oophorectomy (69). Cadmium enhances Lipopolysaccharide (LPS) and interleukin 4 (IL-4) mediated activation. The activation-induced cytosine deaminase (AID) is expressed in B cells via the estrogen receptor (34, 70). It can up-regulate the angiotensin II type 1-receptor-agonistic autoantibodies (AT1-AAs), inducing hypertension (21, 34). Cadmium-mediated complement C5a receptor activates C5, which further activates AT1R and leads to increased blood pressure (34). *In vitro* studies have shown that cadmium up-regulates gene expression in the transforming growth factor-β (TGF-β) pathway and IL-8 (71–73), thereby inhibiting the migration of placental trophoblast cells (73). Reduced migration of trophoblast cells is arguably one of the mechanisms of the development of preeclampsia (74, 75). Cd induces IL-6 production in trophoblast cells through a reactive oxygen species (ROS) dependent activation of the extracellular signal-regulated kinases (ERK)1/2 to and increased ERK1/2, c-Jun N-terminal kinases (JNK), and c-Jun phosphorylation (71), it then stimulates B cell production of AT1R (66, 76), contributing to the endothelial dysfunction and eventually hypertension in pregnancy. Furthermore, normal pregnant rats with long-term injection of IL-6 showed a significant increase in arterial pressure (77). Previous studies have identified that cadmium exposure decreased the expression of vascular endothelial growth factor (VEGF) and placental growth factor (PLGF) (51, 55), which activate systemic maternal endothelia, leading to vascular injury and hypertension (74, 78). *In vivo* studies have suggested that cadmium may induce preeclampsia by impairing the immune function (34), increasing oxidative DNA damage in the placenta (79), and damaging the kidneys (80). However, Sutoo and Akiyama found contradictory facts in rats (81). They observed that exposure to Cd increases dopamine through calmodulin, which in turn lowers blood pressure (81). Whether cadmium raises blood pressure remains controversial.

There was a systematic review that reported the correlation between Cd exposure and preeclampsia. Pollack et al. study in 2014 included only three studies. Two of the investigations were cross-sectional. One did not adjust for possible confounder factors, while the other only reported term preeclampsia in pregnancy outcomes (82). The limited number of articles may give a biased perspective.

By comparison, our report has several strengths. First, this is the first meta-analysis to comprehensively pool blood, urine, and placental Cd to explore the association between Cd and preeclampsia. Second, our meta-analysis included all the recent available studies to avoid selection bias maximally. We also included articles written in Chinese, as many articles focus on this topic. This is because the environmental contamination of heavy metals is a hot research topic in China currently. This helps complete evidence for this research. Third, we conducted meta-regression to further explore the association and determine the source of heterogeneity between studies.

However, we also noted some limitations in our study. The studies that met the inclusion criteria were still limited compared to other heavy metal studies (83), which confined us from exploring deeper. Furthermore, some studies reported the correlation between Cd and preeclampsia but did not report their data completely, and hence, we could not pool them together for further analysis (36, 38). Thirdly, not all studies followed ACOG’s diagnostic criteria. For those which followed ACOG’s, the version of the guidelines may be different. This may impact the study group, causing selection bias in the original study. Lastly, we failed to identify the causes for significant heterogeneity. This might be attributed to the abovementioned reasons, i.e., different study designs, different diagnostic criteria and various measurement methods. Future reviews with more well-designed original studies may alleviate the in-between study heterogeneity.

## 5 Conclusion

In summary, our meta-analysis provides quantitative evidence that Cd exposure is positively associated with preeclampsia in pregnancy. Large cohort studies and animal studies are needed to further clarify cadmium’s role in PE’s pathogenesis.

## Data availability statement

The original contributions presented in the study are included in the article/Supplementary material, further inquiries can be directed to the corresponding author.

## Author contributions

CL: Conceptualization, Investigation, Data curation, Methodology, Software, Writing – original draft. JL: Conceptualization, Data curation, Investigation, Software, Writing – original draft, Formal Analysis. YY: Data curation, Investigation, Software, Methodology, Writing – original draft. QW: Data curation, Investigation, Methodology, Software, Supervision, Validation, Visualization, Writing – review & editing. YZ: Formal Analysis, Investigation, Supervision, Writing – review & editing, Data curation, Project administration, Software, Validation. ZZ: Investigation, Conceptualization, Formal Analysis, Funding acquisition, Supervision, Writing – review & editing.

## Footnotes

^1^https://www.crd.york.ac.uk/prospero/display_record.php?RecordID=361291
