# Supplementary material for: The relationship between cadmium exposure and preeclampsia: a systematic review and meta-analysis
Source: Front Med (Lausanne). 2023 Dec 1;10:1259680. doi: 10.3389/fmed.2023.1259680 (PMC10722428; doi:10.3389/fmed.2023.1259680)
Supplement: Supplementary file 1 [file Data_Sheet_1.docx]

Supplementary Material

## Supplementary Figures

| (A) | (B) |
| --- | --- |

**Supplementary Figure 1.** The Begg’s test and Egger’s test to assess publication bias. (A) Begg’s tests for publication bias. (B) Egger’s test for publication bias.

**Supplementary Figure 2.** The leave-one-out sensitivity analysis to assess publication bias.
