# Supplementary material for: The relationship between cadmium exposure and preeclampsia: a systematic review and meta-analysis
Source: Front Med (Lausanne). 2023 Dec 1;10:1259680. doi: 10.3389/fmed.2023.1259680 (PMC10722428; doi:10.3389/fmed.2023.1259680)
Supplement: Supplementary file 2 [file Data_Sheet_2.docx]

Supplementary Tables

# Supplementary Data

**Search Strategy**

**PubMed**

#1 ("Cadmium"[Mesh]) OR (Cadmium[title/abstract]) OR (Cd[title/abstract]) OR ("heavy metal"[title/abstract]) OR ("trace element"[title/abstract])

#2 (preeclampsia[title/abstract]) OR ("pregnancy hypertension"[title/abstract]) OR ("hypertensive disorders of pregnancy"[title/abstract]) OR ("pregnancy-induced hypertension"[title/abstract]) OR (gestosis[title/abstract]) OR ("gestational hypertension"[title/abstract]) OR ("pregnancy-associated hypertension"[title/abstract]) OR ("pregnancy toxemia"[title/abstract]) OR (eclampsia[title/abstract]) OR (pre-eclampsia[title/abstract]) OR ("HELLP syndrome*"[title/abstract])

#3 #1 AND #2

N=89

**Embase**

#1 Cadmium:ti,ab,kw OR Cd:ti,ab,kw OR 'heavy metal':ti,ab,kw OR 'trace element':ti,ab,kw

#2 preeclampsia:ti,ab,kw OR 'pregnancy hypertension':ti,ab,kw OR 'hypertensive disorders of pregnancy':ti,ab,kw OR 'pregnancy-induced hypertension':ti,ab,kw OR gestosis:ti,ab,kw OR 'gestational hypertension':ti,ab,kw OR 'pregnancy-associated hypertension':ti,ab,kw OR 'pregnancy toxemia':ti,ab,kw OR eclampsia:ti,ab,kw OR pre-eclampsia:ti,ab,kw OR 'HELLP syndrome* ':ti,ab,kw

#3 #1 AND #2

N=100

**Web of Science**

#1 TS=(Cadmium OR Cd OR heavy metal OR trace element)

#2 TS=(preeclampsia OR pregnancy hypertension OR hypertensive disorders of pregnancy OR pregnancy-induced hypertension OR gestosis OR gestational hypertension OR pregnancy-associated hypertension OR pregnancy toxemia OR eclampsia OR pre-eclampsia OR HELLP syndrome*)

#3 #1 AND #2

N=104

**Scopus**

#1 TITLE-ABS-KEY(Cadmium) OR TITLE-ABS-KEY (Cd) OR TITLE-ABS-KEY ("heavy metal") OR TITLE-ABS-KEY ("trace element")

#2 TITLE-ABS-KEY(preeclampsia) OR TITLE-ABS-KEY ("pregnancy hypertension") OR TITLE-ABS-KEY("hypertensive disorders of pregnancy") OR TITLE-ABS-KEY("pregnancy-induced hypertension") OR TITLE-ABS-KEY(gestosis) OR TITLE-ABS-KEY("gestational hypertension") OR TITLE-ABS-KEY("pregnancy-associated hypertension") OR TITLE-ABS-KEY("pregnancy toxemia") OR TITLE-ABS-KEY(eclampsia) OR TITLE-ABS-KEY(pre-eclampsia) OR TITLE-ABS-KEY("HELLP syndrome*")

#3 #1 AND #2

N=372

**CNKI (Chinese National Knowledge Infrastructure)**

CNKI was applied with similar search strategy, using Chinese equivalent words of ‘Cadmium and (pre-eclampsia or eclampsia or hypertensive disorder complicating pregnancy or HELLP syndrome)’ as two key words during search process.

N=27

# Supplementary Tables

**Table 1. Quality assessment using a modified form of Newcastle-Ottawa Scale (NOS) for cross-sectional studies**[1]

| Included studies | Selection | | | | Comparability | Outcome | | Score  (0-10) |
| --- | --- | --- | --- | --- | --- | --- | --- | --- |
|  | ① | ② | ③ | ④ | ⑤ | ⑥ | ⑦ |  |
| Liu 2019[2] | ★ | ★ | ★ | ★ | ★★ | ★ | ★ | 9 |
| Liu 2018[3] | ★ | ★ | ★ | ★ | ★★ | ★ | ★ | 9 |

Selection (maximum five stars) ① Representativeness of the sample; ② Sample size; ③ Non-repondents; ④ Ascertainment of exposure (risk factors, maximum two stars);

Comparability (maximum two stars): ⑤ Comparability of outcomes based on the design or analysis;

Outcome (maximum three stars): ⑥ Assessment of outcome (maximum 2 stars); ⑦ Statistical test

“★” represents requirement fulfilled, and score “1” in this section; “☆” indicates not fulfill the requirement or not stated in the study and hence score “0” in this section.

**Table 2. Quality assessment using Newcastle-Ottawa Scale (NOS) for case-control studies**

| Included studies | Selection | | | | Comparability | Exposure | | | Score  (0-9) |
| --- | --- | --- | --- | --- | --- | --- | --- | --- | --- |
|  | ① | ② | ③ | ④ | ⑤ | ⑥ | ⑦ | ⑧ |  |
| Laine 2015[4] | ★ | ★ | ★ | ★ | ★ | ★ | ★ | ★ | 8 |
| Wang 2018[5] | ★ | ★ | ★ | ★ | ★★ | ★ | ★ | ★ | 9 |
| Bommarito 2019[6] | ★ | ★ | ★ | ★ | ★★ |  | ★ | ★ | 8 |
| Li 2022[7] | ★ | ★ | ★ | ★ | ★★ | ★ | ★ | ★ | 9 |
| Ovayalu 2021[8] | ★ |  | ★ | ★ | ★★ | ★ | ★ | ★ | 8 |
| Wang 2020[9] | ★ | ★ | ★ | ★ | ★★ | ★ | ★ | ★ | 9 |
| Zhang 2018[10] | ★ | ★ | ★ | ★ | ★ | ★ | ★ | ★ | 8 |
| Wang 2008[11] | ★ | ★ | ★ | ★ | ★ | ★ | ★ | ★ | 8 |
| Kosanovic 2002[12] | ★ | ★ | ★ | ★ | ★ | ★ | ★ | ★ | 8 |
| Maduray 2017[13] | ★ | ★ | ★ |  | ★ | ★ | ★ | ★ | 7 |
| Moyene 2016[14] | ★ | ★ | ★ | ★ | ★★ | ★ | ★ | ★ | 9 |
| Kolusari 2008[15] | ★ | ★ | ★ |  | ★ | ★ |  | ★ | 6 |
| Obadia 2018[16] |  |  | ★ | ★ | ★★ | ★ | ★ | ★ | 7 |
| Vigeh 2006[17] | ★ | ★ | ★ | ★ | ★★ | ★ | ★ | ★ | 9 |

NOS for Newcastle-Ottawa Scale

Selection (maximum 4 stars): ① Is the case definition adequate; ② Representative of the cases; ③ Selection of controls; ④ Definition of controls;

Comparability (maximum two stars): ⑤ Comparability of cases and controls based on the design or analysis;

Exposure (maximum three stars): ⑥ Ascertainment of exposure; ⑦ Same method of ascertainment for cases and controls; ⑧ Non-response rate.

**Table 3. Quality assessment using Newcastle-Ottawa Scale (NOS) for cohort studies**

| Included studies | Selection | | | | Comparability | Exposure | | | Score |
| --- | --- | --- | --- | --- | --- | --- | --- | --- | --- |
|  | ① | ② | ③ | ④ | ⑤ | ⑥ | ⑦ | ⑧ |  |
| Yazbeck 2009[18] | ★ | ★ | ★ | ★ | ★ | ★ | ★ | ★ | 8 |

Selection (maximum four stars): ① Representativeness of the exposed cohort; ② Selection of the non-exposed cohort; ③ Ascertainment of exposure; ④ Demonstration that outcome of interest was not present at the start of study;

Comparability (maximum two stars): ⑤ Comparability of cohorts based on the design or analysis;

Outcome (maximum three stars): ⑥ Assessment of outcome; ⑦ Was follow-up long enough for outcomes to occur; ⑧ Adequacy of follow up of cohorts

**Table 4. Preferred Reporting Items for Systematic Reviews and Meta-Analyses: The PRISMA Statement 2009 Checklist.**

| Section/topic | # | Checklist item | Reported on page # |
| --- | --- | --- | --- |
| **TITLE** | | |  |
| Title | 1 | Identify the report as a systematic review, meta-analysis, or both. | 1 |
| **ABSTRACT** | | |  |
| Structured summary | 2 | Provide a structured summary including, as applicable: background; objectives; data sources; study eligibility criteria, participants, and interventions; study appraisal and synthesis methods; results; limitations; conclusions and implications of key findings; systematic review registration number. | 1-2 |
| **INTRODUCTION** | | |  |
| Rationale | 3 | Describe the rationale for the review in the context of what is already known. | 2 |
| Objectives | 4 | Provide an explicit statement of questions being addressed with reference to participants, interventions, comparisons, outcomes, and study design (PICOS). | 2 |
| **METHODS** | | |  |
| Protocol and registration | 5 | Indicate if a review protocol exists, if and where it can be accessed (e.g., Web address), and, if available, provide registration information including registration number. | 3 |
| Eligibility criteria | 6 | Specify study characteristics (e.g., PICOS, length of follow-up) and report characteristics (e.g., years considered, language, publication status) used as criteria for eligibility, giving rationale. | 3 |
| Information sources | 7 | Describe all information sources (e.g., databases with dates of coverage, contact with study authors to identify additional studies) in the search and date last searched. | 3 |
| Search | 8 | Present full electronic search strategy for at least one database, including any limits used, such that it could be repeated. | 3 |
| Study selection | 9 | State the process for selecting studies (i.e., screening, eligibility, included in systematic review, and, if applicable, included in the meta-analysis). | 3-4, Figure 1 |
| Data collection process | 10 | Describe method of data extraction from reports (e.g., piloted forms, independently, in duplicate) and any processes for obtaining and confirming data from investigators. | 3-4 |
| Data items | 11 | List and define all variables for which data were sought (e.g., PICOS, funding sources) and any assumptions and simplifications made. | 3-4 |
| Risk of bias in individual studies | 12 | Describe methods used for assessing risk of bias of individual studies (including specification of whether this was done at the study or outcome level), and how this information is to be used in any data synthesis. | 3-4 |
| Summary measures | 13 | State the principal summary measures (e.g., risk ratio, difference in means). | 4 |
| Synthesis of results | 14 | Describe the methods of handling data and combining results of studies, if done, including measures of consistency (e.g., I^2^) for each meta-analysis. | 4 |
| Risk of bias across studies | 15 | Specify any assessment of risk of bias that may affect the cumulative evidence (e.g., publication bias, selective reporting within studies). | 4 |
| Additional analyses | 16 | Describe methods of additional analyses (e.g., sensitivity or subgroup analyses, meta-regression), if done, indicating which were pre-specified. | 4 |
| **RESULTS** | | |  |
| Study selection | 17 | Give numbers of studies screened, assessed for eligibility, and included in the review, with reasons for exclusions at each stage, ideally with a flow diagram. | 4-5,  Fig 1 |
| Study characteristics | 18 | For each study, present characteristics for which data were extracted (e.g., study size, PICOS, follow-up period) and provide the citations. | Table 1 |
| Risk of bias within studies | 19 | Present data on risk of bias of each study and, if available, any outcome level assessment (see item 12). | Suppl Table 1-3 |
| Results of individual studies | 20 | For all outcomes considered (benefits or harms), present, for each study: (a) simple summary data for each intervention group (b) effect estimates and confidence intervals, ideally with a forest plot. | 5, Fig 2 |
| Synthesis of results | 21 | Present results of each meta-analysis done, including confidence intervals and measures of consistency. | 5-6, Table 1 |
| Risk of bias across studies | 22 | Present results of any assessment of risk of bias across studies (see Item 15). | 5-6, Fig 3, Fig 5, Suppl Fig 1 |
| Additional analysis | 23 | Give results of additional analyses, if done (e.g., sensitivity or subgroup analyses, meta-regression, see Item 16). | 5-6, Fig 3, Fig 5, Suppl Fig 1-2 |
| **DISCUSSION** | | |  |
| Summary of evidence | 24 | Summarize the main findings including the strength of evidence for each main outcome; consider their relevance to key groups (e.g., healthcare providers, users, and policy makers). | 6-7 |
| Limitations | 25 | Discuss limitations at study and outcome level (e.g., risk of bias), and at review-level (e.g., incomplete retrieval of identified research, reporting bias). | 7-8 |
| Conclusions | 26 | Provide a general interpretation of the results in the context of other evidence, and implications for future research. | 7-8 |
| **FUNDING** | | |  |
| Funding | 27 | Describe sources of funding for the systematic review and other support (e.g., supply of data); role of funders for the systematic review. | 8 |

**Reference**

[1] P. A. Modesti, G. Reboldi, F. P. Cappuccio et al., *Panethnic Differences in Blood Pressure in Europe: A Systematic Review and Meta-Analysis.* PLoS One, **11**(1): p. e0147601.2016

[2] T. Liu, M. Zhang, E. Guallar et al., *Trace Minerals, Heavy Metals, and Preeclampsia: Findings from the Boston Birth Cohort.* J Am Heart Assoc, **8**(16): p. e012436.2019

[3] H. Liu, W. Xia, S. Xu et al., *Cadmium body burden and pregnancy-induced hypertension.* Int J Hyg Environ Health, **221**(2): p. 246-251.2018

[4] J. E. Laine, P. Ray, W. Bodnar et al., *Placental Cadmium Levels Are Associated with Increased Preeclampsia Risk.* PLoS One, **10**(9): p. e0139341.2015

[5] F. Wang, F. Fan, L. Wang et al., *Maternal Cadmium Levels During Pregnancy and the Relationship with Preeclampsia and Fetal Biometric Parameters.* Biol Trace Elem Res, **186**(2): p. 322-329.2018

[6] P. A. Bommarito, S. S. Kim, J. D. Meeker et al., *Urinary trace metals, maternal circulating angiogenic biomarkers, and preeclampsia: a single-contaminant and mixture-based approach.* Environ Health, **18**(1): p. 63.2019

[7] X. Li, T. Yu, M. Zhai et al., *Maternal cadmium exposure impairs placental angiogenesis in preeclampsia through disturbing thyroid hormone receptor signaling.* Ecotoxicol Environ Saf, **244**: p. 114055.2022

[8] A. Ovayolu, V. A. Turksoy, I. Gun et al., *Analyses of maternal plasma cadmium, lead, and vanadium levels in the diagnosis and severity of late-onset preeclampsia: a prospective and comparative study.* J Matern Fetal Neonatal Med: p. 1-8.2021

[9] Y. Wang, K. Wang, T. Han et al., *Exposure to multiple metals and prevalence for preeclampsia in Taiyuan, China.* Environ Int, **145**: p. 106098.2020

[10] Q. Zhang, Y. Huang, K. Zhang et al., *Progesterone attenuates hypertension and autoantibody levels to the angiotensin II type 1 receptor in response to elevated cadmium during pregnancy.* Placenta, **62**: p. 16-24.2018

[11] S. Wang, K. Wang, H. Wang, L. Yang, *The association between maternal zinc status and cadmium levels and changes in blood pressure in pregnancy.* Acta Acad Med Mil Tert, **17**: p. 1678.2008

[12] M. Kosanovic, M. Jokanovic, M. Jevremovic, S. Dobric, D. Bokonjic, *Maternal and fetal cadmium and selenium status in normotensive and hypertensive pregnancy.* Biol Trace Elem Res, **89**(2): p. 97-103.2002

[13] K. Maduray, J. Moodley, C. Soobramoney, R. Moodley, T. Naicker, *Elemental analysis of serum and hair from pre-eclamptic South African women.* J Trace Elem Med Biol, **43**: p. 180-186.2017

[14] J. P. Elongi Moyene, H. Scheers, B. Tandu-Umba et al., *Preeclampsia and toxic metals: a case-control study in Kinshasa, DR Congo.* Environ Health, **15**: p. 48.2016

[15] A. Kolusari, M. Kurdoglu, R. Yildizhan et al., *Catalase activity, serum trace element and heavy metal concentrations, and vitamin A, D and E levels in pre-eclampsia.* J Int Med Res, **36**(6): p. 1335-41.2008

[16] P. Musa Obadia, T. Kayembe-Kitenge, V. Haufroid, C. Banza Lubaba Nkulu, B. Nemery, *Preeclampsia and blood lead (and other metals) in Lubumbashi, DR Congo.* Environ Res, **167**: p. 468-471.2018

[17] M. Vigeh, K. Yokoyama, F. Ramezanzadeh et al., *Lead and other trace metals in preeclampsia: a case-control study in Tehran, Iran.* Environ Res, **100**(2): p. 268-75.2006

[18] C. Yazbeck, O. Thiebaugeorges, T. Moreau et al., *Maternal blood lead levels and the risk of pregnancy-induced hypertension: the EDEN cohort study.* Environ Health Perspect, **117**(10): p. 1526-30.2009
